# Expression of *GBGT1* is epigenetically regulated by DNA methylation in ovarian cancer cells

**DOI:** 10.1186/1471-2199-15-24

**Published:** 2014-10-07

**Authors:** Francis Jacob, Megan P Hitchins, André Fedier, Kevin Brennan, Sheri Nixdorf, Neville F Hacker, Robyn Ward, Viola A Heinzelmann-Schwarz

**Affiliations:** 1 Gynecological Research Group, Department of Biomedicine, University Hospital Basel and University of Basel, Hebelstrasse 20, CH-4013 Basel, Switzerland; 2 Adult Cancer Program, Lowy Cancer Research Centre, Prince of Wales Clinical School, University of New South Wales, 2052 Sydney, Australia; 3 Department of Medicine, Stanford School of Medicine, Stanford University, Stanford, CA 94305 USA; 4 Gynaecological Cancer Centre, Royal Hospital for Women, School of Women’s and Children’s Health, 2031 Sydney, Australia

**Keywords:** Globo series, Glycosphingolipids, Forssman antigen, Epigenetics, DNA methylation, Transcriptional regulation

## Abstract

**Background:**

The *GBGT1* gene encodes the globoside alpha-1,3-*N*-acetylgalactosaminyltransferase 1. This enzyme catalyzes the last step in the multi-step biosynthesis of the Forssman (Fs) antigen, a pentaglycosyl ceramide of the globo series glycosphingolipids. While differential *GBGT1* mRNA expression has been observed in a variety of human tissues being highest in placenta and ovary, the expression of *GBGT1* and the genes encoding the glycosyltransferases and glycosidases involved in the biosynthesis of Fs as well as the possible involvement of DNA methylation in transcriptional regulation of *GBGT1* expression have not yet been investigated.

**Results:**

RT-qPCR profiling showed high *GBGT1* expression in normal ovary surface epithelial (HOSE) cell lines and low *GBGT1* expression in all (*e.g.* A2780, SKOV3) except one (OVCAR3) investigated ovarian cancer cell lines, a finding that was confirmed by Western blot analysis. Hierarchical cluster analysis showed that *GBGT1* was even the most variably expressed gene of Fs biosynthesis-relevant glycogenes and among the investigated cell lines, whereas *NAGA* which encodes the alpha-*N*-acetylgalactosaminidase hydrolyzing Fs was not differentially expressed. Bisulfite- and COBRA-analysis of the CpG island methylation status in the *GBGT1* promoter region demonstrated high or intermediate levels of *GBGT1* DNA methylation in all ovarian cancer cell lines (except for OVCAR3) but marginal levels of DNA methylation in the two HOSE cell lines. The extent of DNA methylation inversely correlated with *GBGT1* mRNA and protein expression. Bioinformatic analysis of *GBGT1* in The Cancer Genome Atlas ovarian cancer dataset demonstrated that this inverse correlation was also found in primary ovarian cancer tissue samples confirming our cell line-based findings. Restoration of GBGT1 mRNA and protein expression in low *GBGT1*-expressing A2780 cells was achieved by 5-aza-2’-deoxycytidine treatment and these treated cells exhibited increased *helix pomatia* agglutinin-staining, reflecting the elevated presence of Fs disaccharide on these cells.

**Conclusions:**

*GBGT1* expression is epigenetically silenced through promoter hypermethylation in ovarian cancer. Our findings not only suggest an involvement of DNA methylation in the synthesis of Fs antigen but may also explain earlier studies showing differential *GBGT1* expression in various human tissue samples and disease stages.

## Background

The *GBGT1* gene was first described in canine kidney cells by Haslam *et al.* in 1996 [1] and three years later cloned in human [2]. *GBGT1* mRNA expression has been observed in a broad variety of human tissues including small and large intestines, placenta, and ovary [2]. *GBGT1* encodes the Forssman synthetase (globoside alpha-1,3-*N*-acetylgalactosaminyltransferase 1). Although differential methylation of *GBGT1* has been associated with inflammatory bowel disease [3] and *GBGT1* nonsense and inactivating missense mutations have been identified that produce a truncated or enzymatically inactive enzyme [4], it is not understood how *GBGT1* expression is regulated.

**Figure 1:**
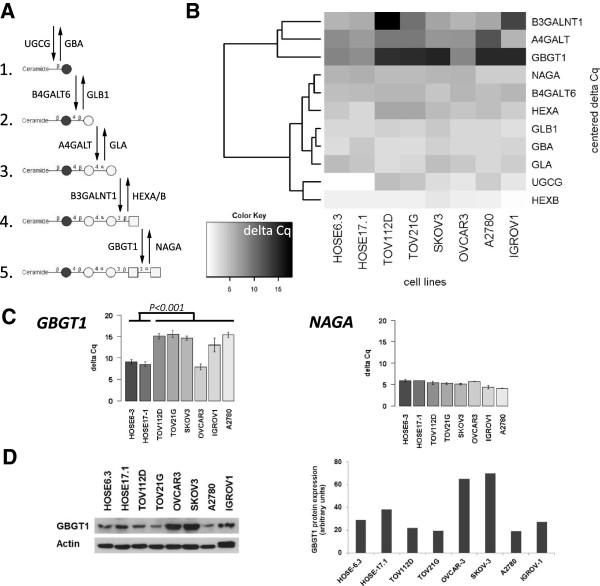
**Expression of glycosyltransferase and glycosidase genes (glycogenes) involved in the biosynthesis of Fs antigen. (A)** Schematic presentation of the biosynthesis pathway of Fs pentasaccharide glycosphingolipid from glycosylceramide and of the responsible glycosyltransferases (downward arrows) and glycosidases (upward arrows): 1. glycosylceramide; 2. lactosylceramide; 3. globotriaosylceramide (Gb3, P^k^); 4. globoside (P antigen, Gb4); 5. Forssman (Fs, IV3GalNAcα-Gb4Cer). Filled circles (glucose, Glc); open circles (galactose, Gal); squares (*N*-acetylgalactosamine, GalNAc). **B)** Heat map with the color key representing normalized (ΔCq) and clustering of glycogene expression (rows) among the investigated cell lines (columns). Color key indicates relative level of expression from high (white) to low (black). Dendrogram shows the result of the hierarchical clustering calculation for the glycogene expression. **(C)** Normalized expression of *GBGT1* and *NAGA*; Mean ± SD (three independent experiments) in the presented cell lines. A high ΔCq value indicates low level of normalized gene expression. **(D)** Western blot analysis (autoradiograph and corresponding quantitative analysis) showing GBGT1 protein expression in the presented cell lines (actin is the sample loading control).

Forssman synthetase catalyzes the last step in the biosynthesis of Forssman (Fs) antigen that involves a series of sequential attachments of monosaccharides catalyzed by the glycosyltransferase UGCG, B4GALT6, A4GALT, B3GALNT1, and finally GBGT1 (Figure 1A). However, it is unknown how and to what extent the expression levels of not only each one of these glycosyltransferases but also of the respective glycosidases such as NAGA (alpha-*N*-acetylgalactosaminidase; hydrolyses Fs antigen) affect the biosynthesis of Fs antigen in the cells. Likewise, it has not yet been investigated whether the expression levels of these glycosyltransferases and glycosidases differ between cancer and normal cells.

Fs antigen is a pentaglycosyl ceramide (GalNAcα1-3GalNAcβ1-3Galα1-4Galβ1-4Glcβ1-1Cer) terminated by GalNAc and belongs to the globo series of glycosphingolipids (GSL). GSL comprise a heterogeneous group of membrane lipids formed by a ceramide (Cer) backbone covalently linked to a glycan moiety: either galactose or glucose to form galactosylceramide (GalCer) or glucosylceramide (GlcCer), respectively. GlcCer is the precursor of at least four different series of GSL. Among those are the globo series GSL which play roles in various biological processes: they are present within lipid raft microdomains [5], they associate with multidrug resistance [6], with angiogenesis [7], and with malignant diseases [8,9], with proliferation [10]. and serve as receptors for human pathogens [11]. Only recently, naturally occurring anti-glycan antibodies against globo series GSL have been identified the levels of which were lower in the plasma of ovarian cancer patients compared to healthy women [9,12,13], an observation that allows discriminating cancer patients from healthy women.

The appearance of Fs antigen is controversial. It was thought to be absent in humans and to be expressed exclusively on animal red blood cells [14]. This is in contrast to studies demonstrating that Fs antigen is present in human cancer [15,16]. *Svensson* and colleagues found the Fs GSL expressed also on human erythrocytes [17].

In the present study we (i) profiled a panel of ovarian cancer and normal ovary surface epithelial cell lines for the expression of *GBGT1*, *NAGA*, and the other glycogenes encoding the glycosyltransferases and glycosidases involved in the biosynthesis of Fs, (ii) investigated whether *GBGT1* expression is regulated through DNA methylation and whether the degree of DNA methylation correlates with *GBGT1* expression in cell lines and tissue, and (iii) determined whether *GBGT1* expression is an outcome predictor in ovarian cancer.

Our results show that *GBGT1* is the most variably expressed gene among the Fs-relevant glycogenes and among the investigated cell lines, DNA methylation is involved in the regulation of *GBGT1* expression in ovarian cancer cell lines and tissue, and *GBGT1* expression does not predict survival.

## Results

### Differential expression of Forssman antigen biosynthesis-relevant glycogenes in ovarian cell lines

We investigated whether the expression level of *GBGT1* differs between ovarian cancer and normal ovarian surface epithelial cells. Because the biosynthesis of Fs antigen requires multiple glycan-processing enzymes, this investigation also includes the glycogenes encoding the corresponding glycosyltransferases and glycosidases. The biosynthesis pathway of Fs pentasaccharide is outlined in Figure 1A. To this aim we profiled the transcriptional activity of these 11 genes in a panel of ovarian cancer cell lines (TOV112D, TOV21G, OVCAR3, SKOV3, A2780, IGROV1) and normal ovarian surface epithelium cell lines (HOSE6-3, HOSE17-1) using RT-qPCR in concordance with MIQE guidelines [18].

The hierarchical cluster analysis of the ΔCq values for each glycogene, portrayed as a heat map, produced two branches (Figure 1B). One branch clustered eight genes (*NAGA*, *B4GALT6*, *HEXA*, *GLB1*, *GBA*, *GLA*, *UGCG*, *HEXB*) with generally comparable expression levels among the cell lines, except for the *UGCG* gene which was expressed at considerably higher levels in both HOSE cell lines compared with the ovarian cancer cell lines. The other branch clustered three genes (*B3GALNT1*, *A4GALT*, and *GBGT1*) with a wide variation in gene expression levels among the cell lines tested. *B3GALNT1* was lowest expressed in TOV112D cells (ΔCq = 17.8), moderately in IGROV1 (ΔCq = 12.9), and highly expressed in the remaining cell lines (ΔCq ranging from 5.5-9.9). *A4GALT* was generally expressed at high levels in all the cell lines (ΔCq ranging from 5.62-9.54), except for A2780 (ΔCq = 11.98). *GBGT1* was highly expressed in both HOSE cell lines (HOSE6-3 ΔCq = 8.86; HOSE17-1 ΔCq = 8.00) and in the OVCAR3 ovarian cancer cell line (ΔCq = 8.74) but expressed at low levels in the remaining cell lines (ΔCqs ranging from 14.44-14.99). Cluster analysis also demonstrated that *GBGT1* branched first in the respective cluster, indicating that *GBGT1* is the most significantly differentially expressed gene in this cluster among the cell lines, being expressed at low levels in ovarian cancer cells (except OVCAR3) and at high levels in normal ovarian surface epithelial cells.

The preferential expression of *GBGT1* in ovarian cancer cell lines was fully confirmed in an additional set of independent experiments (Figure 1C). These results showed that *GBGT1* expression is significantly different among the tested cell lines (*P* < 0.001). *GBGT1* expression is high (indicated by low ΔCq_mean_ ± ΔCq_SD_ values) in both HOSE cells (ΔCq_HOSE6–3_ = 9.07 ± 0.52; ΔCq_HOSE17–1_ = 8.55 ± 0.55) and in OVCAR3 cells (ΔCq_OVCAR3_ = 7.88 ± 0.70) and low in A2780 cells (ΔCq_A2780_ = 15.41 ± 0.62), showing that *GBGT1* expression is 185-times (ΔΔCq) lower in A2780 cells than in OVCAR3 cells. The comparison of the two normal cell lines (HOSE6-3 and HOSE17-1) with the panel of ovarian cancer cell lines (TOV112D, TOV21G, SKOV3, OVCAR3, IGROV1 and A2780) revealed a significantly differential *GBGT1* expression (*P* < 0.001). In contrast to *GBGT1*, *NAGA* (encodes the alpha-*N*-acetylgalactosaminidase that hydrolyses Fs pentasaccharide) was not differentially expressed among the cell lines. Western blot analysis and densitometry data (Figure 1D) demonstrated that HOSE6-3, HOSE17-1 and OVCAR3 cells display higher levels of GBGT1 protein than A2780, TOV112D, TOV21G, and IGROV1 cells. These data were (apart from SKOV3 showing high GBGT1 protein level despite low mRNA levels) largely consistent with the RT-qPCR data.

### Silencing of *GBGT1* expression in ovarian cancer cells by DNA methylation

**Figure 2:**
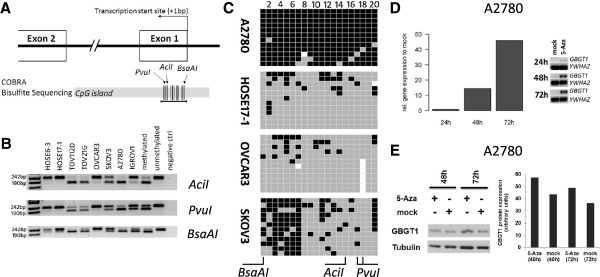
**DNA methylation of *GBGT1 *promoter region. (A)** Illustration of the organization of the CpG island encompassing the transcription start site of the *GBGT1* gene. The horizontal black line represents the 217 bp amplicon generated using COBRA and bisulfite sequencing. Vertical lines represent the organization of individual CpG dinucleotides within the amplified region. Black triangles indicate the respective recognition sites of the restriction endonucleases (*Acil, Pvul and BsaAl*). Each of these enzymes digested the amplicon only when the respective CpG dinucleotide(s) within the enzyme recognition site was methylated in the original DNA prior to bisulfite conversion (*AciI*-active 50 + 176 bp; *PvuI*-active 37 + 180 bp; *BsaAI*-active 192 + 25 bp). **(B)** COBRA with each of the three different endonucleases (*Acil, Pvul, BsaAl*) revealed considerable variation in the methylation status among the cell lines. The degree of methylation (which relates to the intensity of the lower digested bands as compared to the upper undigested band) was consistent between each restriction enzyme for each cell line. **(C)** Methylation profiles of individual CpG sites from single DNA strands derived from bisulfite sequencing in A2780, HOSE17-1, OVCAR3, and SKOV3. Columns represent individual CpG sites. Rows represent number of sequenced clones (n = 12). Methylated CpG (black), unmethylated (grey), unknown status (white). **(D)** Restoration of *GBGT1* expression in A2780 induced by treatment with 2.5 μM 5-Aza. RT-qPCR shows a time-dependent increase in *GBGT1* transcription. Left, data are presented as the number of PCR products in 5-Aza treated samples relative to the mock-treated control (y-axis) as a function of time (24 h, 48 h, 72 h) after treatment (x-axis). Right, RT-qPCR products of *GBGT1* and normalization control *YWHAZ*. **(E)** Western blot (autoradiograph and corresponding quantitative analysis) showing 5-Aza -induced increase in GBGT1 protein expression as a function of time after treatment in A2780.

We next investigated whether the observed differential expression levels of *GBGT1* among cell lines correlated with the DNA methylation status of its CpG island promoter region. Combined bisulfite and restriction analysis (COBRA) was performed to determine the methylation status within a 217 bp fragment of the CpG island located on chromosome 9: 136038417–136039577. This CpG island contains 105 individual CpG sites spanning 1161 bp, according to the UCSC Genome Bioinformatics data base (http://www.genome.ucsc.edu). The targeted genomic DNA sequence for the COBRA assay is located within the CpG island from position +28 bp to +245 bp downstream from the transcription start site (+1 bp) (Figure 2A). The results showed substantial levels of DNA methylation in the three investigated CpG sites in TOV112D, TOV21G, and A2780 cells **(**Figure 2B), consistent with the qPCR and protein data showing significantly reduced *GBGT1* expression (Figure 1C, D). In contrast, no methylation was found in OVCAR3 and in both HOSE cells (Figure 2B) which exhibited high levels of *GBGT1* expression. An intermediate level of methylation was found in SKOV3 and IGROV1 cells (Figure 2B).

Bisulfite sequencing was performed to quantify the *GBGT1* promoter methylation levels in A2780 (high level), HOSE17-1 (low level), OVCAR3 (low level), and SKOV3 (intermediate level), and also to determine the extent of methylation across the CpG island fragment within individual DNA strands. Figure 2C showed that 95.8% of all CpGs are methylated in A2780, whereas only 12.1% were methylated in HOSE17-1 and 9.5% in OVCAR3 cells. The SKOV3 cell line displayed an intermediate 40% methylation of CpGs, preferentially at the first 7 CpG sites. Bisulfite sequencing therefore showed a strong inverse correlation between *GBGT1* expression and DNA methylation (r = −0.86; Pearson correlation), indicating that the majority of ovarian cancer cells have silenced *GBGT1* expression through DNA methylation.

### Increased *GBGT1* expression induced by 5-aza-2’-deoxycytidine treatment

We determined whether silenced *GBGT1* expression in A2780 cells (95.8% methylation) can be reversed by 5-aza-2’-deoxycytidine (5-Aza) treatment. 5-Aza is a DNA methyltransferase inhibitor that de-methylates the DNA and therefore reactivates or enhances the transcription of genes suppressed by DNA methylation [19]. We found that 5-Aza produced a 15-fold increase in *GBGT1* expression after 48 h and a 46-fold increase after 72 h in A2780 cells (Figure 2D), which is also reflected on the GBGT1 protein level (Figure 2E). A 5-Aza-induced effect was also observed for SKOV3 cells (40% methylation) though to a lesser extent (data not shown).

### Elevated HPA-staining in A2780 cells after 5-aza-2’-deoxycytidine treatment

**Figure 3:**
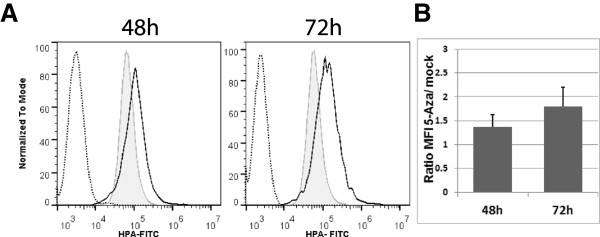
**Elevated HPA-staining in A2780 cells treated with 5-Aza. (A)** Flow cytometry data (representative of three independent experiments) showing a rightwards peak shift (black solid line) after 48 h and 72 h of 5-Aza-treatment relative to the mock control (grey line). The unstained control is shown by the dashed line. **(B)** Plot illustrates the increase in HPA-staining manifested as the increase in the mean fluorescence intensity (MFI) relative to the mock controls as a function of time after 5-Aza -treatment. Data presented as the mean ± SD of three independent experiments.

In order to determine whether the 5-Aza-induced increase in *GBGT1* expression results in elevated presence of Fs antigen on A2780 cells we used HPA (*Helix pomatia* agglutinin)-staining as a “surrogate marker” for Fs antigen. HPA is a lectin which preferentially binds the Fs_di_ disaccharide [20], the terminal structure of the Fs antigen pentasaccharide. Flow cytometry analysis data showed increased HPA-staining in 5-Aza treated A2780 cells (48 h *p* = 0.25; 72 h *p* = 0.08; student *t* test; Figure 3) which reflects the elevated presence of Fs on these cells.

### Differential DNA methylation of *GBGT1* in ovarian tumor and normal adjacent tissue (analysis of The Cancer Genome Atlas (TCGA) data)

**Figure 4:**
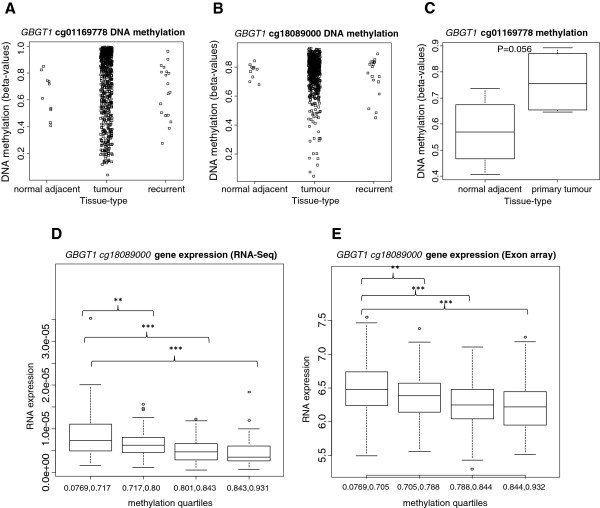
***GBGT1 *methylation and RNA expression levels in The Cancer Genome Atlas (TCGA) ovarian cancer data.** Methylation levels within the *GBGT1* CpG island, detected at the **(A)** cg01169778 and **(B)** cg18089000 probes on the Illumina Infinium HumanMethylation27 BeadChip array in normal-adjacent ovarian tissue (n = 10), primary ovarian tumor (n = 528) and recurrent tumor (n = 33), do not show a difference among these three groups. **(C)** Box-plot showing a borderline significant difference in methylation between matched normal adjacent tissue and primary tumor pairs (n = 4 cases). **(D)***GBGT1* RNA expression, determined by the Illumina HiSeq RNA-Seq platform, stratified by *GBGT1* methylation levels at the cg18089000 probe divided into quartiles, (n = Q1(lowest):67, Q2:64, Q3:64, Q4:70). **(E)***GBGT1* gene expression, determined by the Huex-1_0-st-V2 Affymetrix exon array, stratified by quartiles of methylation levels at the *GBGT1* cg18089000 probe (n = Q1:141, Q2:145, Q3:144, Q4:141). **P-value (linear regression) <0.01, ***P < 0.001.

We wondered whether the observed differential *GBGT1* methylation pattern, i.e. largely unmethylated *GBGT1* in the normal HOSE cells and hypermethylated in ovarian cancer cells, was also found in primary and recurrent ovarian carcinoma and in normal adjacent tissue. To this aim we analyzed the methylation levels of *GBGT1* using the publicly accessible data from The Cancer Genome Atlas (TCGA) ovarian cancer sample set [21]. DNA methylation at two CpG sites within the *GBGT1* CpG island promoter, cg18089000 (located 403 bp upstream of the *GBGT1* transcription start site,) and cg01169778 (located 613 bp downstream of it), were represented on the Illumina Infinium HumanMethylation27 BeadChip array (27 k array). Methylation between these two sites was significantly correlated within primary tumor data (n = 528, Spearman rho = 0.32, *P* = 1.352e-15). Within the TCGA ovarian tissue dataset, DNA methylation data for normal adjacent ovarian tissue were available for a small number (n = 10) of individuals, of which only four had matched tumor. The levels of DNA methylation in ovarian tumors from the TCGA dataset varied widely, which was consistent with our findings in the ovarian cancer cell lines. *GBGT1* methylation levels at both cg01169778 and cg18089000 in adjacent normal ovarian tissues were consistently high (Figure 4A,B). When all normal samples (n = 10) were compared with all primary tumors samples (n = 528), no significant difference in methylation at either cg01169778 (*P* = 0.5694) or cg18089000 (*P* = 0.1136) was found (Figure 4A,B). However, across individuals for which paired tumor and normal-adjacent tissue methylation data was available, cg01169778 was higher in tumors compared with normal adjacent tissue in all four individuals (mean difference = 19.1% between tumors and normal adjacent) and this difference was borderline statistically significant (*P* = 0.056) (Figure 4C). Methylation of cg18089000 was not significantly different between these four matched normal and tumor pairs (*P* = 0.43).

### Association between DNA methylation and expression

We now investigated the dependence of *GBGT1* expression on DNA methylation in tissue samples to translate our cell line-based findings.

Integrated analysis of *GBGT1* DNA methylation and gene expression in the TCGA ovarian cancer data set showed that DNA methylation at cg18089000 was inversely correlated with *GBGT1* expression in primary tumors, measured using either the Huex-1_0-st-V2 Affymetrix exon array (n = 583, rho = −0.27, *P* = 1.869e-11), or the Illumina HiSeq RNA-Seq platform (n = 265, rho = −0.42, *P* = 1.312e-12) (Additional file 1: Table S1). *GBGT1* cg18089000 methylation was not significantly correlated with *GBGT1* exon expression in normal adjacent tissue (n = 10, Spearman rho = 0.55, *P* = 0.10) in this small sample size analysis. In linear models adjusted for age at pathological diagnosis and clinical stage, cg18089000 methylation was significantly associated with *GBGT1* expression, measured by the exon array (n = 571, *P* = 0.0005), and RNA-seq data (n = 265, *P* = 2.05e-05). A dose-effect relationship between cg18089000 methylation and gene expression is apparent when GBGT1 expression is stratified by DNA methylation quartiles (Figure 4D,E). In contrast, cg01169778 methylation did not significantly correlate with *GBGT1* expression or associate in linear models in any sample type using either gene expression measurement (Additional file 1: Table S1).

## Discussion

The present study shows that (i) *GBGT1* is the most differentially expressed glycogene involved in the biosynthesis of Fs antigen and (ii) that *GBGT1* expression is silenced through DNA hypermethylation in ovarian cancer cells and tissue but re-activated by 5-Aza treatment. In contrast GBGT1 (iii) does not correlate with clinical outcome (survival) in ovarian cancer patients. We therefore conclude that *GBGT1* expression is epigenetically regulated in ovarian cancer but that it is not a prognostic predictor for survival.

The major finding from the present study is DNA methylation is one mechanism by which GBGT1 expression is regulated in both ovarian cancer cells and tissue. This is a novel finding and expands on a previous study reporting that *GBGT1* is differentially methylated and associated with inflammatory bowel disease [3]. DNA methylation is one out of at least four epigenetic mechanisms by which eukaryotic organisms regulate the transcription of a significant number of genes, and aberrant hypermethylation of CpG island promoters associated with transcriptional silencing is a frequent occurrence in various types of cancer [22]. Epigenetic silencing is, however, only one mechanism by which *GBGT1* expression is regulated. *GBGT1* nonsense and inactivating missense mutations have been identified that produce a truncated or enzymatically inactive protein [4].

Interestingly, a correlation between *GBGT1* repression and DNA hypermethylation was notably not only found in ovarian cancer cell lines. A bioinformatical analysis of the Ovarian TCGA dataset showed a clear-cut inverse correlation between the degree of methylation at the probe closest to the transcription start site and *GBGT1* mRNA expression levels within the TCGA primary ovarian cancers, although the degree of *GBGT1* methylation varied considerably among samples (ranging from nearly unmethylated to hypermethylated) and levels of *GBGT1* methylation were also detected in adjacent normal ovarian tissue. Notably, however, in the four TCGA cases with matched tumor and normal adjacent tissues higher methylation levels were found in tumor samples compared to normal adjacent tissues, being in concordance with our cell line-based observation. The present study is therefore not only the first one addressing *GBGT1* expression and its regulation in ovarian cancer but also suggest that *GBGT1* is differentially expressed in cancer and healthy conditions i.e. lower in ovarian cancer patients and higher in normal cells. This observation is consistent with a previous study showing *GBGT1* mRNA expression in various tissues [2].

From a clinical point of view it is interesting whether *GBGT1* methylation and/or expression levels associated with clinical outcome parameters such as survival and therefore serve as a prognostic factor. However, our ovarian cancer TCGA dataset analysis did not reveal an association between methylation or expression levels and survival (neither in univariate models nor when age- and stage-adjusted), indicating that *GBGT1* methylation or expression levels are not outcome parameters in ovarian cancer.

*GBGT1* is not the only glycosyltransferase-encoding gene regulated by DNA methylation. *B4GALNT2* and *ST3GAL6* have been found to be epigenetically silenced in colon and gastric cancer cells and shown to be re-expressed after 5-Aza treatment [23]. Both are involved in the biosynthesis of the blood group carbohydrate Sd^a^ (GalNAcβ1-4(NeuAcα2-3)Galβ1-4GlcNAc) which is abundantly present on normal gastrointestinal tract mucosa cells but decreased on gastric cancers [24]. *FUT7* has been shown to be differentially methylated in patients with ulcerative colitis and Crohn's disease [3]. Various genes implicated in the glycosylation of secreted *N*-linked glycoproteins may also be epigenetically regulate because 5-Aza treated ovarian cancer cells displayed altered glycosylation of these proteins [25].

Another important finding is that A2780 cells incubated with 5-Aza exhibited an elevated HPA staining. HPA is a lectin from *Helix pomatia* agglutinin that preferentially binds Fs_di_ (GalNAcα1-3GalNAc-R), the terminal disaccharide (the product of GBGT1) of Fs antigen [20,26,27], and therefore HPA staining may serve as a “surrogate marker” for Fs antigen expression. In this case, this finding together with the 5-Aza-induced increase in *GBGT1* expression suggests that the elevated HPA staining may be a result of the increased *GBGT1* expression and Fs synthesis in these cells. On the other hand, we cannot exclude that 5-Aza also affects the expression of glycogenes other than *GBGT1* that, in turn, produces an “elevated binding” of HPA to glycan structures other than Fs_di_. The latter may explain the relatively high level of HPA-staining in “mock”-treated A2780 cells in the respective experiment.

HPA expression has previously been proposed as a determinant for poor prognosis in breast, gastric, and colon cancer [26,28,29]. Given that elevated HPA staining is a result of increased *GBGT1* expression and that HPA staining is a determinant for poor prognosis also in ovarian cancer, we would expect increased *GBGT1* expression in cancer cells. The observation that *GBGT1* expression is lower by trend in ovarian cancer does neither support nor rules out the idea that the HPA-staining may be a prognostic determinant in ovarian cancer. Earlier studies have suggested Fs antibody titers as disease determinants: high Fs autoantibody titers were found in Graves' disease and Hashimoto's thyroiditis [30] and low were found in patients with cancer of the gastrointestinal tract and breast [31].

## Conclusions

Our results identify *GBGT1* as the most differentially expressed Fs-biosynthesis-relevant glycogene among the cell lines investigated and provide evidence that DNA methylation at its promoter region is one mechanism by which *GBGT1* expression is regulated on the transcriptional level in ovarian cancer. This mechanism may explain the observed differential *GBGT1* expression in earlier studies.

## Methods

### Cell lines

The serous ovarian cancer cell lines OVCAR3, SKOV3, A2780 and IGROV1 were cultured in RPMI 1640 medium, the endometrioid ovarian cancer cell line TOV112D and the clear cell ovarian cancer cell line TOV21G in DMEM, and the (normal) human ovarian surface epithelial cells HOSE6-3 and HOSE17-1 in 1:1 Medium 199:MCDB 105. All media (Sigma-Aldrich Pty. Ltd, Castle Hill, Australia) contained 10% FCS (Sigma-Aldrich Pty. Ltd) and penicillin/streptomycin (Sigma-Aldrich Pty. Ltd). Cells were cultured at 37°C in 5% CO_2_. All cultures were free of mycoplasma (routinely checked as by qualitative PCR using VenorGeM® Mycoplasma Detection Kit, Biocene Pty Ltd, Rozelle, Australia).

### RNA and DNA extraction

To examine the expression of the glycogenes and the reference genes, cell cultures were grown in 6-well plates (NUNC, Thermo Fisher Scientific, Roskilde, Denmark), washed twice with DPBS (Invitrogen Pty Ltd, Thornten, NSW, Australia), and lysed by the addition of lysis buffer directly onto the cells (NucleoSpin RNAII Kit, Macherey&Nagel, Scientifix, Clayton, VIC, Australia). Total RNA was extracted according to the manufacturer’s protocol (NucleoSpin RNAII Kit, Macherey&Nagel). After the elution of total RNA in 50 μl RNase-free water, RNA concentration and the ratios for A_260/230nm_ and A_260/280nm_ were measured (NanoDrop ND-1000, Thermo Fisher Scientific). The integrity of the RNA samples, i.e. the RNA integrity number (RIN) and 28S/18S rRNA ratio, was determined by electropherograms (Agilent Bioanalyzer RNA 6000 Nano).

Genomic DNA was extracted from cell cultures grown in 6-well plates. Cells were lysed with 250 μl lysis buffer (20 mM Tris–HCl, 4 mM Na_2_EDTA, 100 mM NaCl), followed by the addition of 25 μl of 10% (w/v) sodium dodecyl sulfate (SDS). The lysate was transferred into an Eppendorf tube and vigorously vortexed. Then 2.5 μl Proteinase K (Finnzymes, Genesearch Pty Ltd., Arundal, Queensland, Australia) was added and protein digestion was performed for at least 2 h at 55°C. Proteins were then precipitated by adding 200 μl of 5.3 M NaCl, followed by centrifugation at 13’000x*g* for 30 min at 4°C. The supernatant was transferred to a different tube and an equal volume of ice-cold isopropanol was added in order to precipitate the DNA by inverting the tubes several times. After centrifugation for 15 min (13’000x*g*) the DNA was washed with 70% ethanol and re-centrifuged for 10 min. Genomic DNA was dissolved in 10 mM Tris HCl pH 8.5 and the DNA concentration was measured with the NanoDrop ND-1000 (Thermo Fisher Scientific).

All RNA extracts were of high quality and purity: the RNA integrity number (RIN) was 10, the values for A_260/280_ and A_260/230_ ranged from 2.08-2.16 and 1.80-2.21, respectively, and the 28 s/18 s ratio ranged from 1.9-2.3.

### Reverse Transcription (RT) and quantitative Polymerase Chain Reaction (qPCR)

An amount of 500 ng to 1 μg total RNA was reverse-transcribed using the iScript Reverse Transcription Supermix for RT-qPCR according to the manufacturer’s instructions (Bio-Rad Laboratories (Pacific) Pty Ltd, Gladesville, Australia). The complementary DNA (cDNA) was stored at −20°C until further use.

QPCR was performed on the selected glycogenes and reference genes (*HSPCB*, *SDHA*, *YWHAZ*) in order to investigate the relative expression of the glycogenes in the selected cell lines and the patient samples. The variation of reference genes for normalization (mean coefficient of variation of 4.75%) reflecting biological and inter-assay variability was in full concordance with our previous observation on selected reference genes in Jacob *et al.* 2013 [32]. A listing of the accession number, the name of the gene, the sequence of forward and reverse primers, and the amplicon size is presented in Table 1. The reference gene primers were selected according to their specificity to human cDNA and their stable expression pattern. The ‘glyco gene’ primers were designed using QuantPrime [33] (purchased from Sigma-Aldrich Pty. Ltd). QPCR was performed with Stratagene Mx3005® (Integrated Sciences Pty. Ltd, Chatswood, Australia) in 96-well microtiter plates using the 2x SensiFAST™ SYBR Lo-ROX kit (Bioline Pty Ltd, Alexandria, Australia) with low ROX as the reference dye. Optimum reaction conditions were obtained with 400 nM specific sense primer, 400 nM specific antisense primer, RNase/DNase-free water, and cDNA template up to final volume of 20 μl. QPCR setups were based on 10 ng of the previously isolated and reverse-transcribed total RNA. Amplifications were performed starting with a 30 sec enzyme activation at 95°C, followed by 40 cycles of denaturation at 95°C for 5 sec, and an annealing/extension step at 60°C for 30 sec. A melt curve was produced from 65-95°C (continuous measurement). All samples including the negative controls were amplified in triplicates. Mean, standard deviation (SD) and coefficient of variation were calculated. For inter-assay comparability a 0.1 threshold was set for all runs for the determination of the Cq values. Quantification cycle (Cq) values of >36 were excluded from further mathematical calculations, because Cq > 36 do not represent quantitative information of the cDNA/RNA amount and therefore presents the end of the qPCR. All RT-qPCR experiments were performed in compliance to MIQE guidelines [18].

**Table 1:** qPCR primers

**Gene**	**Accession number**	**Gene Name**	**Forward Primer 5’-3’**	**Reverse Primer 5’-3’**	**Amplicon size in bp**
*A4GALT*	NM_017436	α1,4-galactosyl transferase	CGCTGGAGCTAGAGATGGATTTGC	AGCCGACCTTCTTTGCCAACAC	78
*GLA*	NM_000169	α galactosidase A precursor	TCTAATGACCTCCGACACATCAGC	ACACTTCAAAGTTGTCTCCCTGTC	130
*HEXA*	NM_000520.4	β hexosaminidase α chain precursor	TTTGTCACACTTCCGCTGTGAG	ACTCCTGCTCACAGAAGCCTAC	80
*GBA*	NM_001171812	glucosidase, β, acid	ACAGCCACAGCATCATCACGAAC	TGGGACTGTCGACAAAGTTACGC	117
*UGCG*	NM_003358	UDP-glucose ceramide glucosyltransferase	TGTGTTGGATCAAGCAGGAGGAC	AACCTCCAACCTCGGTCAGCTATC	96
*B4GALT6*	NM_004775	UDP-Gal:βGlcNAcβ1,4- galactosyltransferase, polypeptide 6	AGGAGGTCCCTATGGCACTAAC	TCTCTACAGACAGGCCCATTAGTC	89
*GLB1*	NM_001079811	galactosidase, β1, transcript variant 1	TGGCCAGCCATTTCGCTACATC	TGAAAGTTCCAGGGCACATACGTC	135
*HEXB*	NM_000521	β-hexosaminidase β chain precursor	GCAAGTGCTGTTGGTGAGAGAC	GTTGTGCAGCTATTCCACGTTCG	118
*GBGT1*	NM_021996.4	Globoside α-1,3-*N*-acetylgalactosaminyltransferase 1	GCACAAGCTTCAGTGTCCTGTG	TGGCTTCTCCCTCTTGTAGTGC	122
*B3GALNT1*	NM_033169	β1,3-*N*-acetylgalactosaminyl transferase 1	TGCTCTATCACGTGGTGCTCTC	ACGCGAGCCGAAGGTTCTTTAC	62
*NAGA*	NM_000262.2	*N*-acetylgalactosaminidase, α	AGCTTCCAGAGCCCAACACATAC	ACATGTCCCAGCAAGAGCACTG	113
*HSPCB*	NM_007355	Heat shock protein 90 kDa α	TCTGGGTATCGGAAAGCAAGCC	GTGCACTTCCTCAGGCATCTTG	80
*YWHAZ*	NM_001135702	Tyrosine 3-monooxygenase/tryptophan 5-monooxygenase activation protein, zeta polypetide	ACTTTTGGTACATTGTGGCTTCAA	CCGCCAGGACAAACCAGTAT	94
*SDHA*	NM_004168	Succinate dehydrogenase complex, subunit A	TGGGAACAAGAGGGCATCTG	CCACCACTGCATCAAATTCATG	86

The qPCR assay performance for each glycogene and three reference genes (*HSPCB*, *SDHA* and *YWHAZ*) was determined in four consecutive 10-fold dilution steps. The value for PCR efficiency ranged from 97.0% (*GBGT1*) to 107.3% (*HEXA*), and the coefficient of determination (R^2^) was always higher than 0.995, indicating that the qPCR assay performance was reliable (Table 2). An amount of 10 ng RNA was used for each of the gene transcripts in the profiling experiments.The ΔCq values for each glycogene, obtained by normalization against the logarithmic mean of the three reference genes profiled in parallel, and for each cell line were subjected to clustering analysis and presented as a heat map (Figure 1).

**Table 2:** qPCR parameters describing the standard curve for each primer pair on eleven glycogenes and three reference genes

**Gene**	**Slope**	**Intercept**	**Efficiency in %**	**R**^**2**^	**Dilution range**
*UGCG*	−3.1875	21.58	105.9	0.999	10 ng-100 ng
*GBA*	−3.2175	24.48	104.6	0.998	10 pg-100 ng
B4GALT6*	−3.1905	29.01	105.8	0.998	5 pg-50 ng
*GLB1*	−3.1640	24.29	107.0	0.991	1 pg-10 ng
*A4GALT*	−3.3425	28.63	99.1	0.998	10 pg-100 ng
*GLA*	−3.2025	25.75	105.2	0.998	10 pg-100 ng
*B3GALNT1*	−3.2425	27.92	103.4	0.999	10 pg-100 ng
*HEXA*	−3.1575	23.92	107.3	0.999	10 pg-100 ng
*HEXB*	−3.2300	22.51	104.0	0.999	10 pg-100 ng
*GBGT1*	−3.395	32.69	97.0	0.995	100 pg-100 ng
*NAGA*	−3.152	28.36	107.0	0.999	100 pg-100 ng
HSPCB*	−3.250	20.09	103.1	0.998	1 pg-100 ng
YWHAZ*	−3.294	20.35	101.2	0.998	1 pg-100 ng
SDHA*	−3.194	24.64	105.6	0.994	1 pg-100 ng

*Standard curve was established previously [32].

### Bisulfite conversion and COBRA (Combined Bisulfite Restriction Analysis)

Bisulfite conversion was performed using the EZ DNA Methylation-Gold™ Kit according to the manufacturer’s protocol (ZYMO RESEARCH, Integrated Sciences, Pty. Ltd, Chatswood, Australia). An amount of 500 ng genomic DNA was used for bisulfite conversion.

Bisulfite converted genomic DNA was subjected to polymerase chain reaction (PCR) using Platinum® *Taq* DNA Polymerase (Invitrogen Pty. Ltd., Blackburn, Australia). The primers, designed using MethPrimer [34] (purchased from Sigma-Aldrich Pty. Ltd., Castle-Hill, Australia). PCR was set up as follows: 1x Platinum *Taq* reaction buffer, 1 mM MgCl_2_, 100 nM dNTP mix, 400 nM forward (GBGT1_COBRAfor6; 5’-TGT TTG TTT TGT TTT AGG TTT TAT TTG-3’) and reverse primer (GBGT1_COBRArev6; 5’-AAA ACC CTC CTC CTT AAC CCC TTA C-3’) and 0.05 units of *Taq* polymerase made up with Ultra Pure™ distilled water (Invitrogen Pty. Ltd., Blackburn, Australia) to a final volume of 20 μl. PCR was performed by initial denaturation at 94.0°C for 5 min, followed by 40 cycles of 94°C for 30 sec, 58°C for 30 sec, 72°C for 45 sec, and finally by amplicon elongation at 72°C for 10 min. The PCR product (size 217 bp) was visualized in a 1.7% agarose gel. Restriction fragment length analysis was performed on PCR products incubated with the restriction endonucleases *AciI* (methylated 50 bp + 167 bp, unmethylated 217 bp), *PvuI-HF* (methylated 37 bp + 180 bp, unmethylated 217 bp) and *BsaAI* (methylated 192 bp + 25 bp, unmethylated 217 bp) (New England BioLabs Inc., Genesearch Pty Ltd., Arundal, Queensland, Australia). Genomic DNA isolated from human blood serum served as an unmethylated COBRA control. Genomic DNA from the same sample treated with CpG methyltransferase *M.SssI* (New England BioLabs Inc., Genesearch Pty Ltd.) served as the positive methylation control.

### Bisulfite Sequencing

COBRA-based PCR for *GBGT1* was performed as described above. For subsequent ligation, the PCR products were extracted from the 1.7% agarose gel using the QIAquick® Gel Extraction Kit (Qiagen Pty Ltd, Doncaster, VIC, Australia) and ligated into pGEM®-T Easy Vector System I according to the manufacturer’s protocol (Promega, Alexandria, NSW, Australia). Afterwards, the entire ligation was heat shock transformed into *E.coli* XL10 GOLD strain. Bacterial cells were held together with the ligation on ice for 30 min, followed by incubation at 42°C for 35 sec. Immediately after heat shock, bacterial cells were placed on ice for 2 min and pre-warmed medium (Sigma-Aldrich Pty. Ltd., Castle-Hill, Australia) was added, followed by incubation at 37°C for 1 h. Transformed bacteria were plated on LB agar plates containing 50 μg/ml carbenicillin, IPTG, and X-Gal, and were incubated overnight at 37°C. White colonies were picked and colony PCR was performed using the T7 universal primer (5’-TAA TAC GAC TCA CTA TAG GG-3’) and SP6 universal primer (5’-ATT TAG GTG ACA CTA TAG-3’) (Sigma-Aldrich Pty) to amplify the plasmid inserts. The conditions were as follows: 1x Platinum *Taq* reaction buffer, 3 mM MgCl_2_, 100nM dNTP mix, 400nM forward and reverse primer, and 0.05 units of *Taq* polymerase made up with Ultra Pure™ distilled water up to a final volume of 20 μl. PCR samples were separated on a 1.7% agarose gel. Free dNTPs were removed by enzymatic PCR clean-up and the remaining primers were incubated with the PCR products for 30 min at 37°C with 2.5U Antarctic phosphatase and 10U Exonuclease I (New England BioLabs Inc., Genesearch Pty Ltd., Arundal, QLD, Australia) dissolved in 1× Antarctic Phosphatase Reaction buffer. The reaction was heat-inactivated at 80°C for 20 min. The PCR clean-up reaction was applied to the sequencing reaction, containing 0.5 μl BigDye®, Terminator v3.1 Cycle Sequencing Kit (Applied Biosystems Australia Pty Ltd, Mulgrave, VIC, Australia), 2 μl 5× sequencing reaction buffer, 0.35 μl SP6 universal primer, and 2.5 μl distilled water. The sequencing reaction was performed using the following conditions: 94°C for 20 sec, 50°C for 20 sec, and 60°C for 4 min. All steps were repeated for 25 cycles. DNA was precipitated by 100% ethanol and 3 M sodium acetate pH5.2, washed with 70% ethanol, and air-dried. Samples were sequenced (ABI 3730 Capillary Sequencer, The Ramaciotti Centre for Gene Functional Analysis, University of New South Wales, Sydney, NSW, Australia). Bisulfite sequencing data were visualized and analyzed using the web based software BISMA, Bisulfite Sequencing Data Presentation and Compilation [35].

### 5-aza-2’-deoxycytidine treatment

Cells carrying a hypermethylated *GBGT1* gene were treated with 5-Aza (Sigma-Aldrich Pty. Ltd; stock solution prepared in 50% (v/v) acetic acid) in order to restore or increase *GBGT1* expression. An amount of 10^5^ cells were seeded in 6-well plates (NUNC, Thermo Fisher Scientific Pty. Ltd., Scoresby, Australia) and treated with 5-Aza (2.5 μM final concentration) on the next day. The culture medium was removed every 24 h and replaced by fresh culture medium containing 2.5 μM 5-Aza. Samples were harvested after 24 h, 48 h and 72 h of treatment. Genomic DNA and total RNA were extracted as described above. Mock controls contained 50% (v/v) acetic acid at concentrations identical to 5-Aza.

### Preparation of cell lysates and Western blot analysis

Whole cell lysates were obtained from subconfluent cultures. The cultures were treated with 5-Aza or a corresponding concentration of acetic acid (mock control) 24 hours after seeding. Cells were then harvested 48 h or 72 h after treatment. Cells were lysed for Western blot analysis according to standard laboratory protocols. The protein concentration of cell lysates was determined by the BCA Protein Assay Kit (Pierce, Perbio Science, Lausanne, Switzerland). Twenty μg protein was loaded and separated using SDS-PAGE, followed by blotting onto a polyvinylidene difluoride membrane (Amersham Biosciences, Otelfingen, Switzerland). Proteins were detected with specific primary antibodies (goat anti-GBGT1, Lab Force, Nunningen, Switzerland; anti-mouse β-actin, Sigma-Aldrich, Buchs, Switzerland; anti-rabbit tubulin, Cell Signaling, BioConcept, Allschwil, Switzerland) and the appropriate horseradish peroxidase-conjugated secondary antibodies (anti-goat IgG-HRP, Lab Force, Basel, Switzerland; anti-rabbit IgG-HRP and anti-mouse IgG-HRP, Cell Signaling, BioConcept, Basel, Switzerland). Complexes were visualized by enhanced chemiluminescence (Amersham Biosciences, Otelfingen, Switzerland) and autoradiography. Densitometry (quantitative analysis of the intensity of the complexes on the autoradiograph, normalized against actin orv tubulin) was performed using the XXXXX software.

### Flow cytometry analysis

The presence of Fs_di_ on the cell membrane was analyzed by flow cytometry (BD Accuri C6 flow cytometer, BD Bioscience, Basel, Switzerland). Biotin-conjugated *helix pomatia* agglutinin (HPA; Sigma-Aldrich Pty. Ltd., Castle-Hill, Australia) followed by streptavidin conjugated to FITC (BD Bioscience, Basel, Switzerland) was used for fluorescence detection. Single cells were gated to quantify HPA-positive cells (cell debris excluded). Data acquisition was performed using BD Accuri C6 software (BD Bioscience, Basel, Switzerland). Data analysis was performed using the FlowJo v10 software (Tree Star Inc., Ashland, USA).

### TCGA clinical sample data analyses

The Cancer Genome Atlas ovarian cancer molecular and clinical data was downloaded from the Broad Institute TCGA Data and Analysis website (https://confluence.broadinstitute.org/display/GDAC/Home) which provides data subjected to standardized preprocessing and normalization, as described in detail on their website.

DNA methylation was measured using the Illumina Infinium HumanMethylation27 BeadChip array (n = 582 primary ovarian tumor sample, 10 normal adjacent tissue samples and 18 recurrent tumor samples). *GBGT1* expression was measured using the Huex-1_0-st-V2 exon array (n = 583 primary tumors overlapping with DNA methylation data) and Illumina HiSeq RNA-Seq (n = 265 primary tumors overlapping with DNA methylation data) platforms. Technical analysis parameters were as described on the TCGA website (http://cancergenome.nih.gov/).

### Statistical analysis

Basic R packages and R package ‘qPCR’ [36] from the open source statistical programming language R (http://CRAN.R-project.org/, version 2.13.2) were used for data analysis of qPCR (including the calculations of PCR efficiency, slope, intercept and r squared (r^2^)). The one-way ANOVA test with a significance level of *P* < 0.01 was applied to compare gene expression among the cell lines. Student’s t test was applied to compare gene expression levels between normal and cancer cell lines. Pearson correlation (R package “stats”) was performed for the relationship between RT-qPCR data and bisulfite sequencing data.

For TCGA data analyses, the Shapiro-Wilks test was used to test for normal distribution within molecular data. DNA methylation deviated significantly from a normal distribution at *GBGT1* cg01169778 (*P* < 2.2e-16) and cg18089000 (*P* < 2.2e-16), and RNA-Seq data for *GBGT1* was not normally distributed (Shapiro Wilks *P* = 8.538e-16), therefore data for these variables were log-transformed for all parametric tests, whereas exon array data, which was not abnormally distributed (*P* = 0.09), was not log transformed. Spearman correlations were used to test for correlations between DNA methylation and gene expression, and linear regression was used to test for associations between methylation and expression, adjusting for other variables, with methylation treated either as a continuous variable or as a categorical variable based on methylation quartiles. The Student’s t-test was used to test for DNA methylation differences between tumor and adjacent normal tissue, with matching for matched pairs. Cox proportional hazard models were used to test for associations between DNA methylation (or gene expression) and survival time, measured as days until death from pathological diagnosis, with methylation treated either as a continuous variable or as a categorical variable based on methylation quartiles. Cox models were adjusted for age at pathological diagnosis and clinical stage, as these factors were significantly associated with survival time (age: *P* = 0.0007, HR = 1.02, stage: *P* = 0.0008, HR = 1.3). Kaplan-Meier plots were used to visually compare survival time differences between DNA methylation categories.

## Competing interests

The authors have declared that they have no competing interests.

## Authors’ contributions

Conceived and designed the experiments: FJ MH AF KB SN VHS; performed the experiments: FJ, AF, SN; analyzed the data: FJ MH SN VHS; contributed reagents/ materials/ analysis tools: FJ MH NH RW VHS; drafted and wrote the manuscript: FJ AF MH VHS. All authors read and approved the final manuscript.

## Supplementary Material

Additional file 1: Table S1 Association between DNA methylation and gene expression in the TCGA ovarian cancer dataset. Click here for file
