# Supplementary material for: Expression of GBGT1 is epigenetically regulated by DNA methylation in ovarian cancer cells
Source: BMC Mol Biol. 2014 Oct 7;15:24. doi: 10.1186/1471-2199-15-24 (PMC4193910; doi:10.1186/1471-2199-15-24)
Supplement: Additional file 1: Table S1 — Association between DNA methylation and gene expression in the TCGA ovarian cancer dataset. [file 1471-2199-15-24-S1.docx]

**Additional file 1: Table S1 Association between DNA methylation and gene expression**

| DNA methylation *versus* expression (Exon array) | | | | | | |
| --- | --- | --- | --- | --- | --- | --- |
| CpG site | Sample_type | N | Spearman Rho | Spearman *P* | GLM *P*** | GLM *P* (adj)*** |
| *GBGT1* Cg18089000 | Normal-adjacent | 10 | 0.55 | 0.1 | _ | _ |
|  | Primary | 571 | -0.27 | 1.87E-11 | 1.29E-06 | 0.0005 |
|  | Recurrent | 33 | -0.26 | 0.15 | _ | _ |
| *GBGT1* Cg01169778 | Normal-adjacent | 10 | 0.52 | 0.13 | _ | _ |
|  | Primary | 571 | 0.078 | 0.06 | 0.85 | 0.87 |
|  | Recurrent | 33 | 0.06 | 0.74 | _ | _ |
| DNA methylation *versus* expression (RNA-Seq)h | | | | | | |
| *GBGT1* Cg18089000 | Normal-adjacent | 0 | _ | _ | _ | _ |
|  | Primary | 265 | -0.42 | 1.31E-12 | 4.22E-08 | 2.05E-05 |
|  | Recurrent | 10 | -0.75 | 0.013 | _ | _ |
| GBGT1 Cg01169778 | Normal-adjacent | 0 | _ | _ | _ | _ |
|  | Primary | 265 | -0.09 | 0.13 | 0.407 | 0.44 |
|  | Recurrent | 10 | 0.06 | 0.15 | _ | _ |
| *Linear regression was performed only for primary tumors due to inadequate sample size in normal tissue and recurrent tumor | | | | | | |
| **General Linear Model |  |  |  |  |  |  |
| ***General linear model  *P*-value adjusted for age ad diagnosis and clinical stage | | | | |  |  |
